# Heat Perception and Aversive Learning in Honey Bees: Putative Involvement of the Thermal/Chemical Sensor AmHsTRPA

**DOI:** 10.3389/fphys.2015.00316

**Published:** 2015-11-25

**Authors:** Pierre Junca, Jean-Christophe Sandoz

**Affiliations:** Evolution, Genomes, Behavior and Ecology, CNRS, Univ. Paris-Sud, IRD, Université Paris-SaclayGif-sur-Yvette, France

**Keywords:** insects, thermoreception, nociception, aversive learning, AmHsTRPA

## Abstract

The recent development of the olfactory conditioning of the sting extension response (SER) has provided new insights into the mechanisms of aversive learning in honeybees. Until now, very little information has been gained concerning US detection and perception. In the initial version of SER conditioning, bees learned to associate an odor CS with an electric shock US. Recently, we proposed a modified version of SER conditioning, in which thermal stimulation with a heated probe is used as US. This procedure has the advantage of allowing topical US applications virtually everywhere on the honeybee body. In this study, we made use of this possibility and mapped thermal responsiveness on the honeybee body, by measuring workers' SER after applying heat on 41 different structures. We then show that bees can learn the CS-US association even when the heat US is applied on body structures that are not prominent sensory organs, here the vertex (back of the head) and the ventral abdomen. Next, we used a neuropharmalogical approach to evaluate the potential role of a recently described Transient Receptor Potential (TRP) channel, HsTRPA, on peripheral heat detection by bees. First, we applied HsTRPA activators to assess if such activation is sufficient for triggering SER. Second, we injected HsTRPA inhibitors to ask whether interfering with this TRP channel affects SER triggered by heat. These experiments suggest that HsTRPA may be involved in heat detection by bees, and represent a potential peripheral detection system in thermal SER conditioning.

## Introduction

In associative learning, animals associate sensory stimuli or their own behavioral responses with particular outcomes, possessing a positive or negative hedonic value for the animal. In classical (or Pavlovian) learning, an initially neutral stimulus such as an odor, sound or color (conditioned stimulus–CS) is associated with a salient appetitive or aversive outcome, like the presence of food or of a noxious stimulus (unconditioned stimulus—US; Pavlov, 1927). Learning success critically depends on the salience of the involved stimuli for the animal, especially on the subjective intensity of the US (Rescorla, 1988; Hammer, 1993; Scheiner et al., 2005). Understanding Pavlovian conditioning therefore implies a careful analysis of how a particular US is detected at the sensory level and how its information is processed within the animal brain.

In honeybees, both appetitive and aversive conditioning can be studied in laboratory conditions thanks to two dedicated protocols (Giurfa and Sandoz, 2012; Tedjakumala and Giurfa, 2013). The conditioning of the proboscis extension response (PER), in which bees associate an odor CS with a sucrose US, is a well-established assay that mimics the final part of bees' foraging behavior, when they experience a floral aroma together with nectar. It has been used for decades for unraveling the neural mechanisms of appetitive learning (Bitterman et al., 1983; Menzel, 1999; Giurfa and Sandoz, 2012). In this paradigm, data are already available about how the sucrose US is detected and processed in the bee brain. Sucrose is detected by dedicated sugar receptors (AmGr1) on gustatory neurons within specific sensilla on the bees' antennae, mouthparts and tarsi (de Brito Sanchez, 2011; Jung et al., 2015). These neurons project to the subesophageal ganglion, where they are thought to directly or indirectly contact a single octopaminergic neuron, VUM-mx1 (ventral unpaired median neuron 1 of the maxillary neuromere), which represents the appetitive reinforcement in the bee brain (Hammer, 1993). It converges at multiple sites with the olfactory pathway, allowing the formation of the odor-sucrose association (Menzel, 1999, 2012).

By contrast, very little information is yet available concerning US detection and perception in aversive conditioning. The most influential aversive learning paradigm is based on the bees' sting extension response (SER). This response represents the final stage of bees' aggressive response to the presence of a potential intruder in front of the hive (Breed et al., 2004), classically elicited by many sensory stimuli (dark colors, moving objects, etc., Free, 1961) and by honeybees' alarm pheromones (Free, 1987). In fixed bee in the laboratory, SER is triggered by noxious stimuli, such as an electric shock (Núñez et al., 1983) or a strong tactile contact (Zhang and Nieh, 2015). In the initial version of the aversive conditioning, bees learned to associate an odor CS with an electric shock US (Vergoz et al., 2007; Roussel et al., 2009). As the electric shock is an unnatural stimulus for bees, a recent study proposed a modified version of SER conditioning, in which the electric shock is replaced by a thermal stimulation with a heated probe as US (Junca et al., 2014). Heat is a natural stimulus for bees and temperature variations play an important role in the life of honeybees. At the colony level, bees strictly regulate the hives' temperature, as deviations from normal brood temperature results in increased mortality as well as in morphological and behavioral defects (Himmer, 1927; Koeniger, 1978; Tautz et al., 2003; Groh et al., 2004; Jones et al., 2005). High temperatures are critical, and in summer, when temperatures rise above the thermal optimum of the hive (~34°C), workers stand at the hive entrance and fan their wings to decrease in-hive temperature. Foragers also bring water inside the hive, thereby cooling air temperature (Lindauer, 1954). At the individual level, bees strictly avoid temperatures above 44°C and respond with a sting extension to heat stimulations (Junca et al., 2014). They thus perceive a high temperature as an aversive stimulus, and can associate an odorant with such a heat stimulus.

Changing the nature of the aversive reinforcement has opened new possibilities for studying US detection and processing. Contrary to the electric shock, which requires using EEG gel and does not easily allow topical applications, the heated probe can be used for precisely stimulating particular parts of the bees' body. In the appetitive modality, US perception varies according to which structure is stimulated with sucrose: mouthparts, antennae and foreleg tarsi (Marshall, 1935; Scheiner et al., 2004; de Brito Sanchez et al., 2008). Several studies have dissected the differential contributions of these potential USs in appetitive olfactory learning (Bitterman et al., 1983; Sandoz et al., 2002; Scheiner et al., 2005; Wright et al., 2007; de Brito Sanchez et al., 2008). First, these studies showed that all three locations support some level of conditioning, although sucrose solution applied to the proboscis leads to higher acquisition success compared to antennal or tarsal USs. This effect is thought to be related to the mouthparts' higher sensitivity to sucrose compared for instance to the tarsi (de Brito Sanchez et al., 2008). In addition, the location of the sucrose US can have an effect on the duration of memory retention and the types of memories produced (Wright et al., 2007). PER conditioning with an antenna-only US supports, shorter memory retention (<24 h) than when bees receive the US on the mouthparts (>96 h; Wright et al., 2007). Thus, different US locations may support different learning and/or retention performances. Sucrose detection is limited to a few structures on the bee body, which have evolved to arbor gustatory sensory organs involved in appetitive behaviors. In aversive learning, by contrast, bees learn to associate an odor with a noxious stimulus, potentially leading to an injury. Contrary to the detection of food stimuli, animals must be able to avoid injuries on their whole body. Until now, we showed that thermal stimulation of the antennae, mouthparts and foreleg tarsi all trigger SER and can act as aversive US, yielding a similar learning success (Junca et al., 2014). In the present study, we asked if in bees, the aversive thermal US must be detected by dedicated sensory organs to act as US (as in appetitive conditioning) or if thermal detection is a more general sensory ability and heat applied anywhere on their body may act as US.

The use of heat as US may also allow searching for the involved peripheral receptors. In the animal kingdom, a wide range of receptors belonging to very different families have been shown to be responsible for temperature detection, from cold to extreme heat (Clapham et al., 2001). Among them, Transient Receptor Potential (TRP) channels seem to be especially important (Montell et al., 1985; Clapham, 2003; Voets et al., 2005). In invertebrates, Drosophila possesses several types of TRP channels involved in high temperature detection. Among them, members of the TRPA subfamily are essential for responding to heat, like Painless and dTRPA1 (Tracey et al., 2003; Hamada et al., 2008; Kwon et al., 2010; Neely et al., 2011). Unfortunately, no *TRPA1* receptor is known in honeybees and *AmPain* is poorly described (Matsuura et al., 2009). However, honey bees express HsTRPA, a Hymenoptera-specific non-selective cationic channel belonging to the TRPA subfamily and activated by temperatures above 34°C (honeybee gene: *AmHsTRPA*, Kohno et al., 2010). When expressed in a heterologous system, this channel's current response increases rather monotonically with increasing temperature without showing any maximum at least until 42°C (it was not tested for higher temperatures). Such response is reminiscent of the SER probability increase observed from room temperature until 65°C in worker bees (Junca et al., 2014). To this day, *HsTRPA* thus represents the best candidate for thermal detection involved in aversive thermal conditioning. This TRP channel is a joint thermal and chemical sensor, being also triggered by exogenous activators like AITC (allyl isothiocyanate), CA (cinnamaldehyde) and camphor (Kohno et al., 2010). Two exogenous inhibitors, Ruthenium Red (RuR) and menthol have also been isolated (Kohno et al., 2010). The existence of both activators and inhibitors for this receptor provides us with the opportunity to test whether HsTRPA is necessary and/or sufficient for thermal detection assessed through SER.

In this study, we first mapped thermal responsiveness all over the honeybee body, by measuring workers' SER after applying heat on 41 different structures. We, then, assessed the aversive olfactory conditioning performances of bees when applying the thermal US on body structures that are not prominent sensory interfaces, the vertex (back of the head) and the ventral abdomen. We next used a neuropharmalogical approach to evaluate the role of HsTRPA for heat detection. First, we performed topical applications of HsTRPA activators on the bee to assess if it is sufficient for triggering SER. Second, we injected HsTRPA inhibitors to ask whether interfering with this TRP channel affects SER triggered by heat.

## Materials and methods

### Animals

Experiments were performed on honey bees caught on the landing platform of several hives on the CNRS campus of Gif-sur-Yvette, France. After chilling on ice, bees were harnessed in individual holders so that both sting- and proboscis extension could be clearly monitored in the same harnessed position. Bees were fed with 5 μl of sucrose solution (50% w/w) every morning to standardize satiety levels and were conserved in a dark and humid box between experiments.

### Stimulations

Thermal stimulations were provided for 1 s by means of a pointed copper cylinder (widest diameter: 6 mm; length: 13 mm), mounted onto the end of a minute soldering iron running at low voltage (HQ-Power, PS1503S). Temperature at the end of the cylinder was controlled using a contact thermometer (Voltcraft, Dot-150). Sucrose stimulations were provided for 1 sec with a soaked toothpick to the bees' antennae.

### Thermal sensitivity map of the bee body

**Figure 1:**
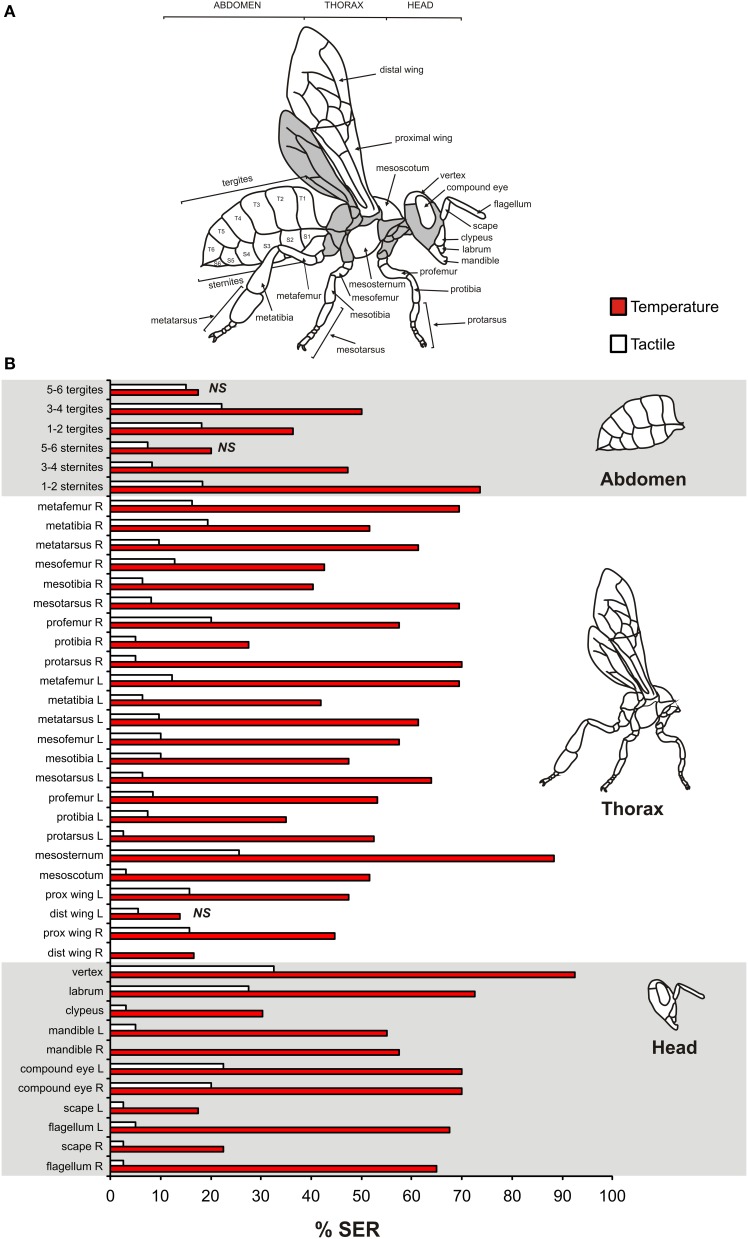
**Impact of thermal stimulation of 41 structures of the honeybee body on sting extension responses (SER). (A)** Map of the bee body showing the names of the tested structures. Gray areas were not accessible in our holding setups and were thus not tested. **(B)** Percentage of SER observed for thermal stimulations on the 41 different body parts using a heated copper probe (*n* = 555, four structures tested per bee). As control, tactile stimulations with an unheated probe were given. Prox, proximal; dist, distal; L, Left; R, Right.

We first aimed at determining whether noxious thermal stimulation of the bees' different body parts triggers a SER and if thermal sensitivity varies among them. Thermal stimulations (65°C for 1 s) were applied on 41 different areas of the bees' body (see Figure 1A). Although, bees' encounters with such a high temperature would be very rare in natural conditions, this stimulation was chosen in order to study bees' thermal nociceptive system. Recent studies in Drosophila have shown that insects possess a nociceptive system which quickly and strongly responds to potentially deadly temperatures and allows them to avoid such stimuli (Tracey et al., 2003; Neely et al., 2011). Our previous work already showed that a short (1 s) stimulation at this temperature triggers clear SER responses when applied on the antennae, the mouthparts or the forelegs of the bees, without inducing any long-lasting effect on bees (Junca et al., 2014). Eleven median unpaired structures were tested: labrum, clypeus, back of the head, mesoscotum, mesosternum, 1-2, 3-4 sternites, 5-6 sternites, 1-2 tergites, 3-4 tergites, 5-6 tergites. Fifteen paired body parts were also tested on the left or right side independently: antenna flagellum, antenna scape, compound eye, mandible, proximal forewing, distal forewing, protarsus, protibia, profemur, mesotarsus, mesotibia, mesofemur, metatarsus, metatibia, metafemur. To avoid any fatigue of the bees, only four structures were tested per bee. In addition to thermal stimulations, tactile controls were applied on the same structures to verify that sting extension was a consequence of thermal stimulation. Tactile stimulations were performed with a duplicate copper probe which remained at ambient temperature. For each bee, the order of stimulation of the different structures, as well as whether each stimulation was performed with the heated or with the control probe, were determined randomly prior to starting the experiment. The eight stimulations were performed at 10 min intervals. In this experiment, two groups of 20 bees were tested each day.

### SER conditioning with a thermal US on the vertex and the ventral abdomen

To assess whether or not bees are able to perform aversive olfactory conditioning with a thermal US on body parts that do not correspond to sensory organs, SER conditioning experiments were carried out with a thermal stimulus (65°C) on 3-4 sternites or on the back of the head as reinforcement. In a differential aversive conditioning procedure, one odorant (the CS+) was associated with a thermal reinforcement (the US), while another odorant was presented without reinforcement (the CS−). The odor CSs were 2-octanone and nonanal (Sigma Aldrich, Deisenhofen, Germany). Five microliters of pure odorant were applied onto a 1 cm^2^ piece of filter paper which was transferred into a 20 ml syringe (Terumo, Guyancourt, France) allowing odorant delivery to the antennae. Half of the honeybees received thermal reinforcement when 2-octanone (odor A) was presented and no reinforcement when nonanal (odor B) was presented, while the reversed contingency was used for the other half of the bees. Both groups were conditioned along 16 trials (8 reinforced and 8 non-reinforced) in which odorants were presented in a pseudo-random sequence (e.g., ABBABAAB) starting with odorant A or B in a balanced way across animals. The inter-trial interval (ITI) was 10 min. Each conditioning trial lasted 36 s. The bee was placed in the stimulation site in front of the air extractor, and left for 18 s before being exposed to the odorant paired with the US. Each odorant (CS+ or CS−) was delivered manually for 4 s. The thermal stimulus started 3 s after odorant onset and finished with the odorant (1 s temperature stimulation). The bee was then left in the setup for 14 s and was then removed. The temperature of 65°C was chosen for the US because this stimulation induced a high rate of SER in the previous experiments. One group of 16 bees was tested daily.

### HsTRPA involvement in thermal sting extension response

We investigated the putative involvement of the thermal/chemical sensor HsTRPA in heat sensitivity as measured by sting extension. To this end, we evaluated the effects of known HsTRPA activators and inhibitors. We focused on the SER triggered by thermal stimulation on the mouthparts, as this is the US commonly used for aversive thermal conditioning (Junca et al., 2014; Cholé et al., 2015).

In a first experiment, we asked if topical application of a chemical HsTRPA activator on the mouthparts directly triggers SER, as a thermal stimulation does. Kohno et al. (2010) isolated three exogenous molecules able to activate this channel: allyl isothiocyanate (AITC), cynnalmaldehyde (CA), and camphor (Sigma Aldrich, Deisenhofen, Germany). These compounds were applied with a soaked toothpick at two concentrations per drug in distilled water: AITC (1 mM and 100 mM), CA (1 mM and 100 mM), camphor (3 and 300 mM). As controls, thermal stimulation (65°C) as above and a toothpick soaked with distilled water (vehicle) were applied to the mouthparts. Activator solutions and controls were provided in a randomized order with a 10 min interval. Two groups of 18 bees divided in three subgroups for each activator were tested each day.

We also evaluated the effect of injections of HsTRPA inhibitors on SER triggered by heat. A small hole was pricked into the cornea of the median ocellus to allow the insertion of a 1 μl microsyringe (Hamilton company, Reno, Nevada, USA). Different groups of bees were injected with 1 μl Ringer solution, menthol in Ringer, or ruthenium red (RuR) in Ringer (Sigma Aldrich, Deisenhofen, Germany). Two concentrations were tested for each drug: menthol (0.5 and 5 mM), RuR (0.1 and 1 mM). One hour after the injections (Kohno et al., 2010), bees received a thermal stimulation (65°C) and a tactile control on the mouthparts, in a randomized order for each bee. Stimulations were performed at 10 min intervals. In a further experiment, bees were injected with the highest inhibitor concentrations (RuR 5 mM or menthol 1 mM) or Ringer and were then subjected to a thermal responsiveness experiment (Junca et al., 2014). One hour after inhibitor injection, bees received a succession of six stimulations of increasing temperature (from ambient temperature ~25 to 75°C), in steps of 10°C. Thermal stimulations alternated with tactile controls, provided as above with an identical unheated probe. Stimulations were applied during 1 s and the bees' SER was noted.

We also verified that the application of HsTRPA inhibitors did not have any non-specific deleterious effects on bees' behavioral responsiveness. We thus chose to assess their potential effect on bees' PER responses to sucrose. After injections with the inhibitors (RuR 5 mM, menthol 1 mM) or Ringer, we performed a typical sucrose responsiveness protocol as described in Scheiner et al. (2004). Bees were presented sucrose solutions of increasing concentration, following an exponential progression (0, 0.1, 0.3, 1, 3, 10, 30% w/w). Sucrose stimulations were alternated with water controls. Sucrose and water stimulations were provided with a soaked toothpick to the bees' two antennae simultaneously, and the PER (extension or not of the proboscis) was noted.

Each trial lasted 38 s. One bee at a time was placed in the setup, and left for 20 s before stimulus application started. The stimulation lasted for 1 s. The bee was then left in the setup for 17 s before being removed. For a given bee, all stimulations were performed at 10 min intervals.

### Statistical analysis

All recorded data were dichotomous, with a sting or proboscis extension being recorded as 1 and a non-extension as 0. When comparing the responses of the same bees to thermal and tactile stimulations on the different structures composing the heat sensory map, pairwise McNemar comparisons were used. Differences in thermal or in tactile responses among body structures were assessed using a Chi^2^ test. When comparing responses to thermal or tactile stimuli across wider areas (lateralization, core/periphery, body parts), Chi^2^ tests were used. For pairwise comparisons, as body parts were composed of three structures (head, thorax, abdomen), each structure was involved in two comparisons. A Bonferroni correction for multiple comparisons was thus applied, and the significance threshold was α_corr_ = 0.05∕2 = 0.025. When analyzing within group the effect of topical applications of HsTRPA activators, McNemar tests were used to compare drug application to water control. To compare between groups the responses of bees injected with HsTRPA inhibitors or vehicle, Fisher's exact test were used. As three groups were involved, the significance threshold was corrected for multiple comparisons as α_corr_ = 0.025. To analyze thermal and sucrose responsiveness curves or aversive conditioning curves, we used repeated measure ANOVAs with stimulus (thermal vs. tactile, sucrose vs. water or CS+ vs. CS−) and trial as repeated factors. For aversive conditioning, following standard procedures, only bees which responded to the US at least three times in the course of acquisition were kept for analysis (vertex: 2%; 3-4 sternites: 29%). To test the effect of inhibitors on thermal and sucrose responsiveness, thermal, or sucrose response curves were compared using repeated measure ANOVAs with drug as a between-group factor. Monte Carlo studies have shown that it is permissible to use ANOVA on dichotomous data only under controlled conditions, which are met in these experiments (Lunney, 1970). Statistical tests were performed with STATISTICA 5.5 (Statsoft, Tulsa, USA).

## Results

### Thermosensory map of the bee body assessed by sting extension

We first aimed to map the heat sensitivity of the different parts of the honeybee body, by applying a heated probe and measuring sting extension responses (SER). Heat was applied for 1 s, and heat stimulations were alternated with tactile controls in a pseudo-randomized order. In total, 41 different structures were tested (Figure 1A, four structures tested per bee, *n* = 555 bees). Figure 1B presents the percentage of responses obtained for each structure to heat and to the tactile control. The proportion of SER to heat stimulation varied among tested structures (Chi^2^ test: Chi^2^ = 235.7, *P* < 0.001, 40 df, from 13.9% SER for the left distal wing to 92.5% SER for the dorsal part of the head (vertex)). Likewise, responses to tactile control stimulations varied according to the tested structure (Chi^2^ test: Chi^2^ = 104.8, *P* < 0.001), from 0% SER (right mandible and right distal wing) to 32% SER (vertex). Overall, 38 out of the 41 tested structures exhibited significantly higher responses to heat than to the tactile control (McNemar test: Chi^2^ > 4.17, *p* < 0.05; exceptions: left distal wing, 5.6 sternites, 5.6 tergites: Chi^2^ < 1.78, NS).

**Figure 2:**
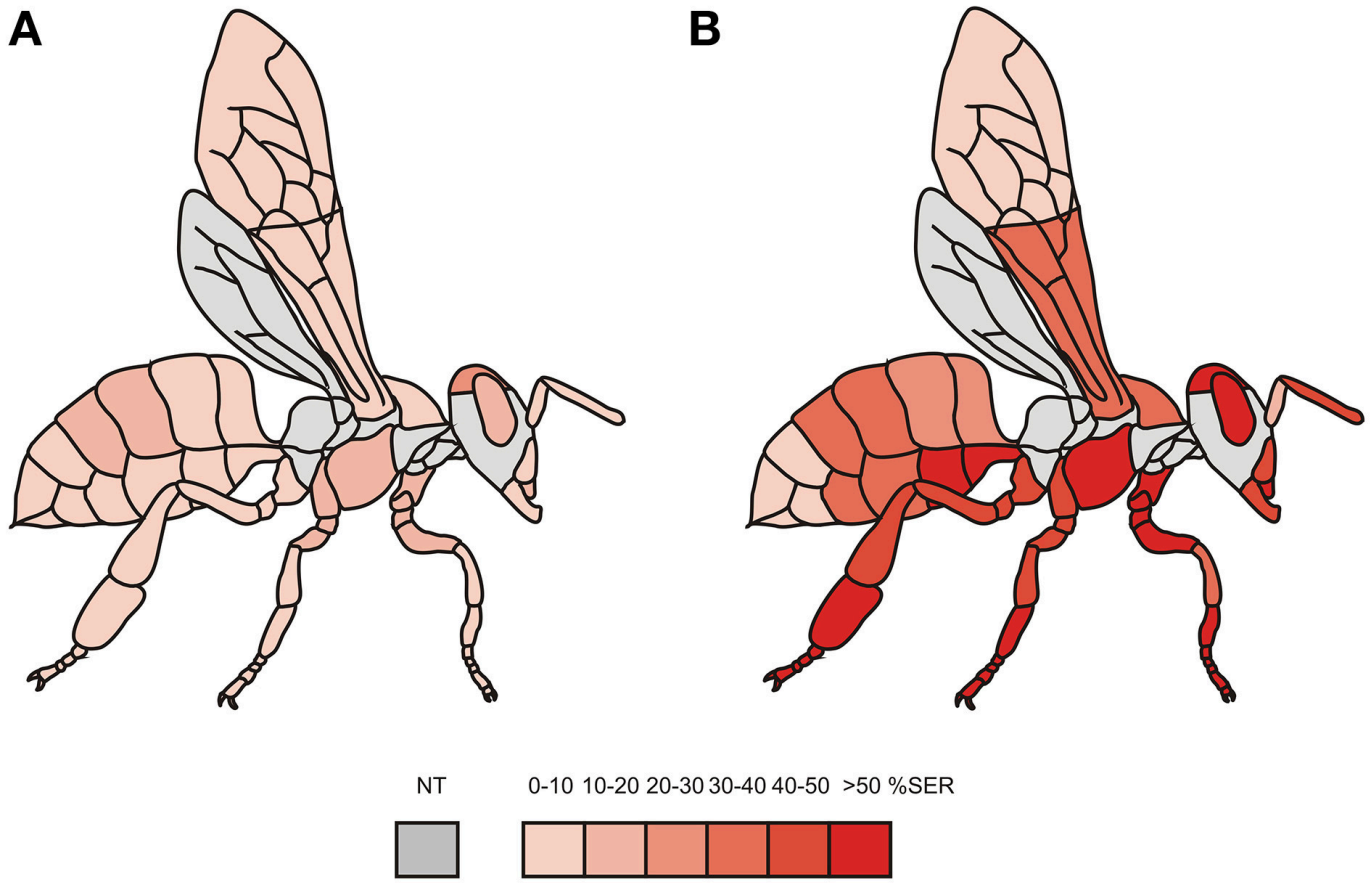
**Tactile and heat sensitivity maps obtained by measuring sting extension responses**. The maps represent the percentage of SER observed after tactile **(A)** or thermal **(B)** stimulation of each structure of the bee body, using a color scale from light red (0–10% of SER) to dark red (>50% of SER). Gray areas were not accessible in our holding setups and were thus not tested (NT). Sting extensions are mainly due to thermal input as seen from the comparison of both maps.

**Figure 3:**
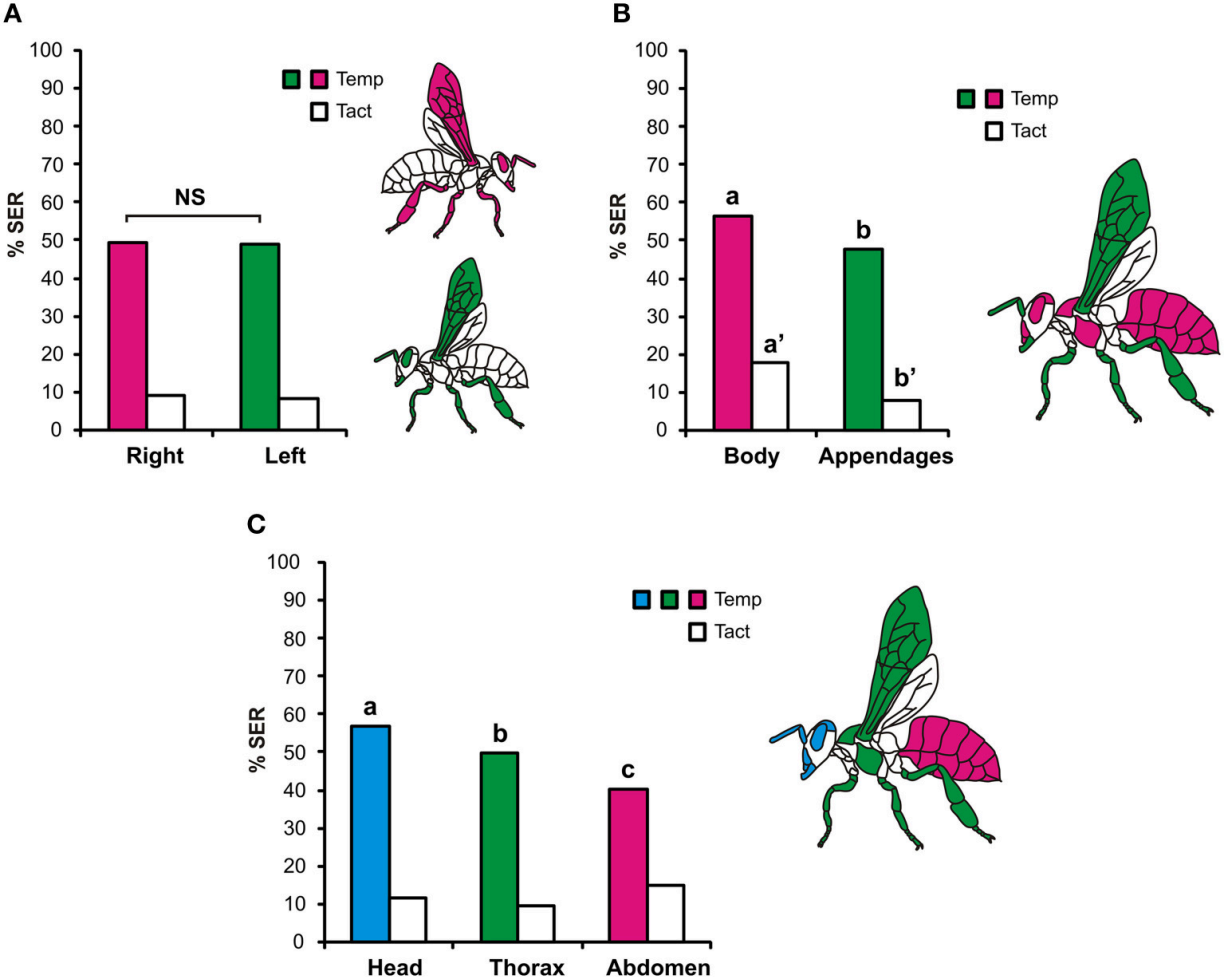
**Tactile and heat sensitivity according to the location of the structures. (A)** Bilateral symmetry: responses of left (green) or right (magenta) structures were pooled and compared. Stimulations on both sides induced similar SER rates. **(B)** Body/appendages: data were pooled for all appendages (green: antennae, mouthparts, legs, wings) and for main body parts (magenta). Body structures responded significantly more than appendages to both tactile and heat stimulations. **(C)** Heat sensitivity according to the antero-posterior axis: data were pooled separately for head (blue), thorax (green), and abdomen (magenta). A gradient of thermal response intensity was found from head to abdomen. Different letters indicate significant differences in Chi^2^ tests.

Figure 2 presents the same data on a schematic individual, using a color scale from light red (0–10% of SER) to dark red (>50% of SER). This map shows strong variations in the responses of the different body parts to heat stimulations, more so than for tactile stimulations. To evaluate this observation statistically, we next analyzed the responses of different body parts according to their localization (Figure 3). First, we asked whether bees' tactile and heat sensitivities are lateralized (Figure 3A). We found that responses to tactile and to heat stimuli were identical between the bees' left and right appendages (tactile: Chi^2^ = 0.10, 1 df, NS; temperature: Chi^2^ = 0.04, 1 df, NS). Second, we asked if a difference in sensitivity exists between the honeybees' body and its different appendages (Figure 3B). We found that SER were significantly more frequent when stimulating the body than when stimulating the appendages, both for thermal stimulation (Chi^2^ = 10.1, 1 df, *p* < 0.01) and for tactile stimulation (Chi^2^ = 35.4, 1 df, *p* < 0.001). Lastly, we examined tactile and heat sensitivity according to the bees' antero-posterior axis (Figure 3C). A significant heterogeneity appeared among body parts (head, thorax, abdomen) in the bees' responses to thermal stimuli (Chi^2^ = 14.4, 2 df, *p* < 0.001) but not to tactile stimuli (Chi^2^ = 5.40, 2 df, NS). Thermal responses were highest for the head (56.8% SER) and lowest for the abdomen (40.4% SER), and all body parts differed from the others (head/thorax: Chi^2^ = 5.99, *p* < α_corr_ = 0.025; head/abdomen: Chi^2^ = 15.9, *p* < α_corr_ = 0.025; thorax/abdomen: Chi^2^ = 6.39, *p* < α_corr_ = 0.025). We thus conclude that although the whole honeybee body is sensitive to thermal stimuli, differences in thermal sensitivity appear among body parts.

### Thermal aversive reinforcement on main body structures

If honey bees are able to detect heat on their whole body and to respond with a SER, one may then wonder whether such stimulations may also act as an aversive reinforcement in a conditioning procedure. Our previous work showed that heat application on the antennae, the mouthparts or the front legs may operate as aversive reinforcement in olfactory SER conditioning (Junca et al., 2014). These structures are however all known sensory organs, acting as interfaces between the animal and its environment. Here, we chose two structures, the rear part of the head (vertex) and the ventral abdomen (3-4 sternites), which are not dedicated sensory structures, and asked whether 65°C stimulations of these structures can act as reinforcement in a differential olfactory conditioning procedure. In this protocol, bees had to differentiate between an odor associated with the thermal stimulation (CS+) and an explicitly non-reinforced odor (CS−).

**Figure 4:**
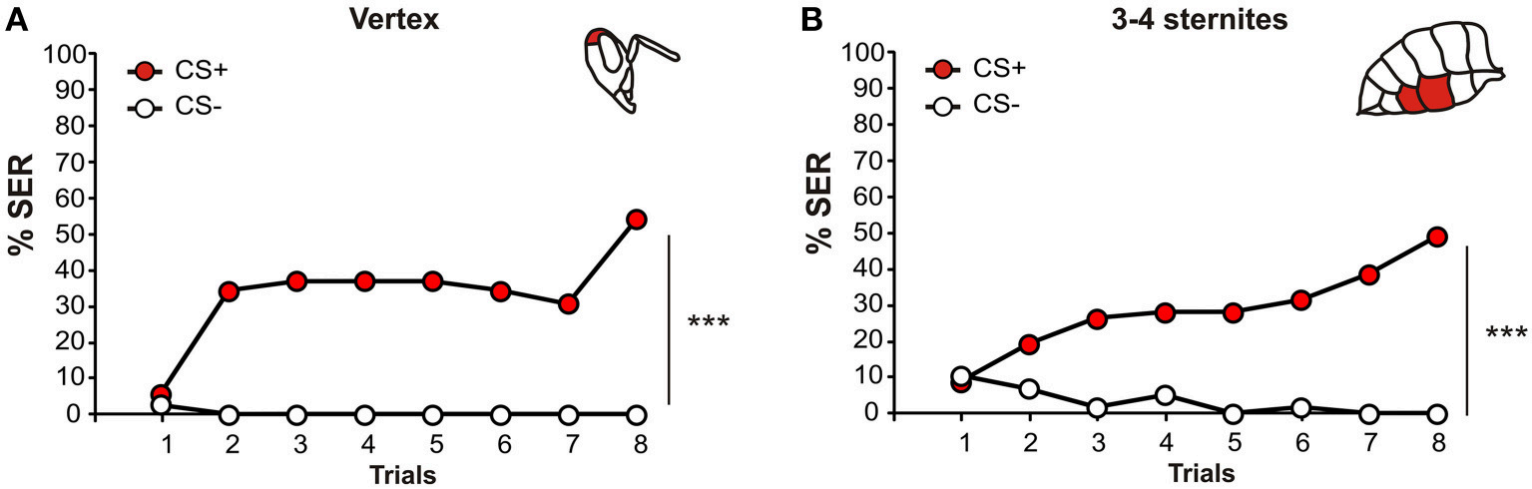
**Thermal aversive conditioning with US application on the head and the abdomen**. Differential olfactory SER conditioning with a US consisting in thermal stimulation of **(A)** the rear of the head (vertex) or **(B)** the ventral abdomen (3-4 sternites). In both cases, honey bees managed to differentiate between the CS+ (red dots) and the CS− (white dots) along the 8 trials (^***^p < 0.001).

Bees learned the task efficiently in both situations (Figure 4). When the vertex was stimulated (Figure 4A, *n* = 37), bees' SER to the CS+ increased significantly (from 6 to 54%, ANOVA for repeated measurements—RM-ANOVA, *F*_(7, 238)_ = 4.13, *p* < 0.001), while their responses to the CS- remained low and stable (*F*_(7, 238)_ = 0.27, NS). Consequently, bees' responses to the CS+ and CS− developed differently in the course of training (stimulus × trial interaction: *F*_(7, 238)_ = 3.89, *p* < 0.001). When the 3-4 sternites were stimulated (Figure 4B, *n* = 57), bees' SER to the CS+ increased along trials (from 9 to 49%, *F*_(7, 392)_ = 5.99, *p* < 0.001) while responses to the CS− did not change throughout the experiment (*F*_(7, 392)_ = 1.81, NS). Accordingly, bees' responses to the CS+ and CS− developed differently in the course of training (stimulus × trial interaction: *F*_(7, 392)_ = 7.66, *p* < 0.001). These results, obtained on the vertex and the ventral abdomen, suggest a general ability of bees to associate odorants (CS) with thermal stimulations on their body (US).

### Impact on SER of topical applications of HsTRPA activators

The previous experiments showed that bees perceive a heat stimulus on their whole body and can use this information in the context of aversive conditioning. But how does heat detection take place at the peripheral level? We focused on HsTRPA, so far the only well-described thermal receptor in the honey bee. As a previous study isolated chemical activators of this receptor *in vitro* (Kohno et al., 2010), we first wondered if topical application of these chemicals is sufficient for triggering a SER. We thus evaluated the effect caused by the application on the bees' mouthparts of a toothpick soaked with AITC (allyl isothiocyanate), CA (cinnamaldehyde) or camphor, in three groups of animals. We focused here on the mouthparts because thermal stimulation of this structure is routinely used in our aversive conditioning experiments (Junca et al., 2014; Junca et al. in preparation). As controls, identical stimulations with a water-soaked toothpick (solvent control) and a heated copper probe (65°C, positive control) were applied. Stimulations were given at 10 min intervals in a randomized order. Two concentrations of each drug were tested.

**Figure 5:**
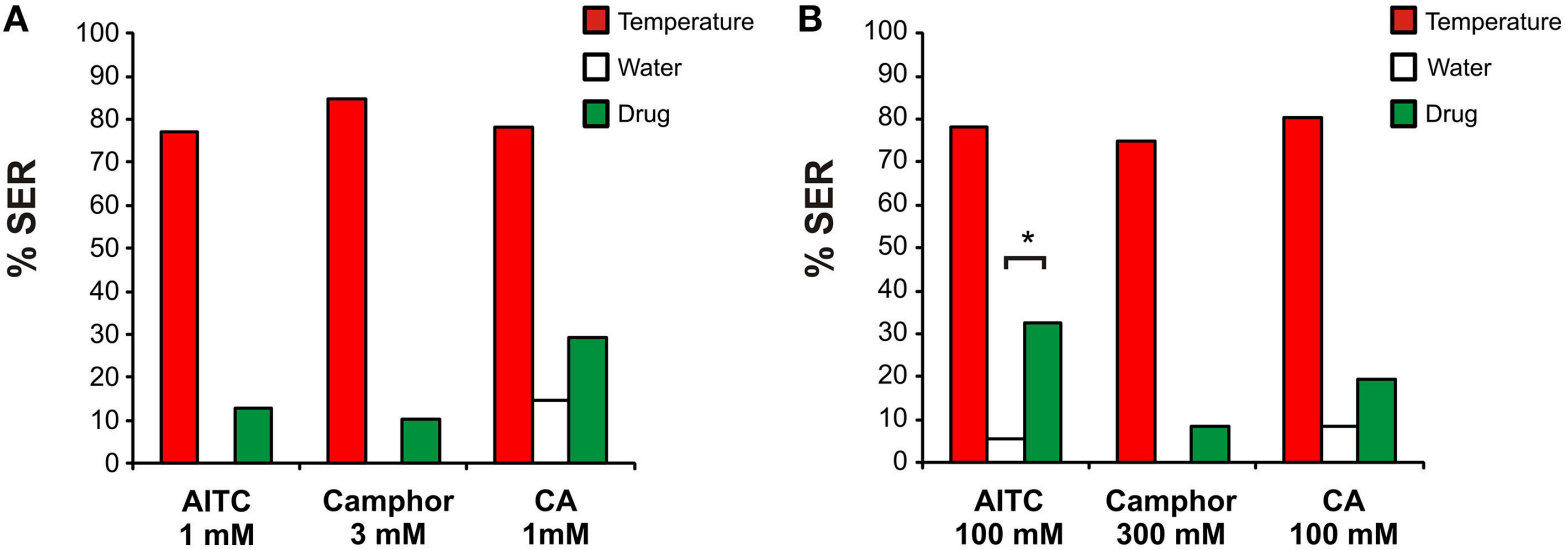
**Effect of topical application of HsTRPA activators on sting extension responses**. The bees' mouthparts were stimulated with AITC (allyl isothiocyanate), CA (cinnamaldehyde) or camphor at two concentrations, **(A)** 1–3 mM or **(B)** 100–300 mM (all drugs in green). A thermal stimulation (red) or a water control (white) were used as controls. Only 100 mM AITC led to significant SER compared to the water control (^*^p < α_corr_ = 0.025).

At the lower concentrations (Figure 5A; 1 mM AITC, *n* = 39; 1 mM CA, *n* = 39; 3 mM camphor, *n* = 41), no effect of the drugs was observed. As expected, honey bees exhibited high SER to the heated probe and low responses to the water control stimulation, with a clear difference between both stimulations (Mc Nemar test, Chi^2^ > 24.04, *p* < α_corr_ = 0.025). However, drugs generally induced low response rates, which were not statistically higher than the water control (Mc Nemar test, Chi^2^ < 3.20, NS). At the 100 times higher concentrations (Figure 5B; 100 mM AITC, *n* = 37; 100 mM CA, *n* = 36; 300 mM camphor, *n* = 36), one of the three drugs was effective in triggering SER. As above, in all groups, thermal stimulation led to strong responses but the water control did not (Mc Nemar test, Chi^2^ > 24.04, *p* < 0.025). While CA and camphor application did not elicit any clear response (Mc Nemar test, Chi^2^ < 1.50, NS), AITC induced 32% SER, which was significantly higher than the water control (Mc Nemar test, Chi^2^ = 8.10, *p* < 0.025). We thus conclude that only one HsTRPA activator was effective when applied topically on the bees' mouthparts, and only at a very high concentration.

### Impact of HsTRPA inhibitors on heat sensitivity

We then asked whether HsTRPA is necessary for bees to detect heat and respond with a sting extension. We focused here on SER triggered by thermal stimulation on the mouthparts, the US commonly used for aversive thermal conditioning (Junca et al., 2014; Cholé et al., 2015). Two chemical inhibitors of HsTRPA have been identified *in vitro* (Kohno et al., 2010), menthol and ruthenium red (RuR). If drug injections provoke a decrease in SER triggered by heat, it would position HsTRPA as a good candidate for high temperature detection. To test this hypothesis, three groups of bees received an injection of 1 μl menthol, RuR, or Ringer (vehicle) as a control, in the median ocellus. After 1 h, bees were then subjected to a thermal stimulation (65°C) to the mouthparts and a tactile control at 10 min intervals in a randomized order. Two concentrations of each drug were tested.

**Figure 6:**
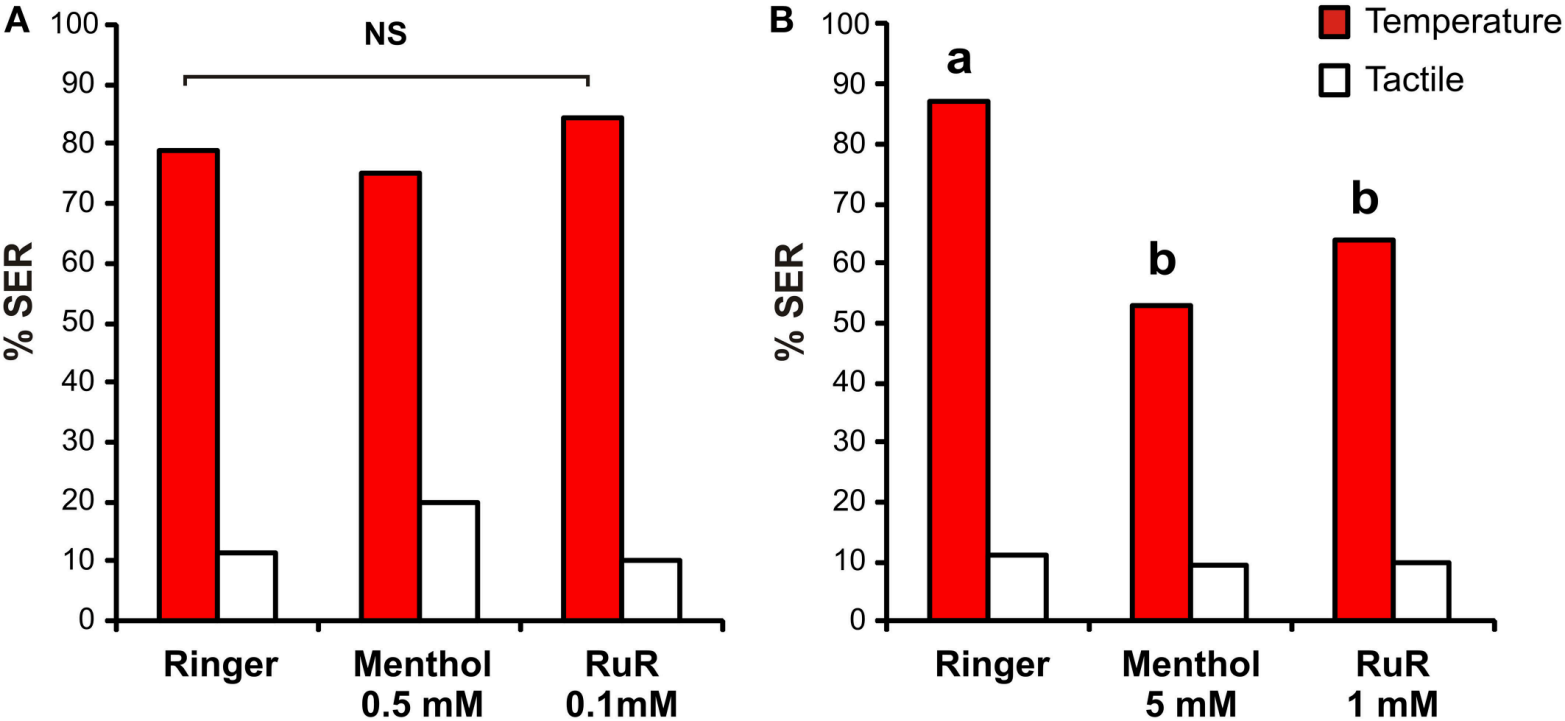
**Impact of HsTRPA inhibitors on SER to thermal stimulations**. Bees were injected in the median ocellus with menthol, ruthenium red (RuR) or Ringer as control. Sting extensions were recorded in response to 1 sec thermal stimulation (65°C; red) and tactile stimulation (white). **(A)** At low concentration (0.5 mM menthol and 0.1 mM RuR), no effect of the inhibitors appeared. **(B)** At 10 times higher concentrations (5 mM menthol and 1 mM RuR) both drugs significantly inhibited SER responses to heat. Different letters indicate significant differences among groups (*p* < α_corr_ = 0.025).

When the lower concentrations of inhibitors were tested (Figure 6A; 0.5 mM menthol, *n* = 40; 0.1 mM RuR, *n* = 39; Ringer *n* = 43), no effect was observed. In all three groups, honey bees exhibited high SER to the heated probe and low responses to the tactile control, with a clear difference between these stimulations (Mc Nemar test, Chi^2^ > 20.0, *p* < 0.001). No difference was observed among groups in SER to the thermal stimulation (Chi^2^ = 1.13, 2 df, NS) or to the tactile control (Chi^2^ = 1.86, 2 df, NS). At the 10 times higher concentration (Figure 6B; 5 mM menthol, *n* = 64; 1 mM RuR, *n* = 61; Ringer *n* = 62), both drugs were effective in blocking SER. Although, in all three groups responses induced by thermal stimuli were still significantly higher than responses to tactile controls (Mc Nemar test, Chi^2^ > 26.0, *p* < 0.001), SER to the heat stimulus was different among groups (Chi^2^ = 17.4, 2 df, *p* < 0.001). In particular, responses to heat were lower in both drug-injected groups compared to the Ringer control group (Fisher's exact test, RuR: Chi^2^ = 8.95, *p* < α_corr_ = 0.025; menthol: Chi^2^ = 17.3, *p* < 0.025). RuR- and menthol-injected groups displayed comparable rates of SER to the thermal stimulus (Fisher's exact test, Chi^2^ = 1.5, NS). No difference appeared among groups in SER to the tactile stimulus (Chi^2^ = 0.14, 2 df, NS).

**Figure 7:**
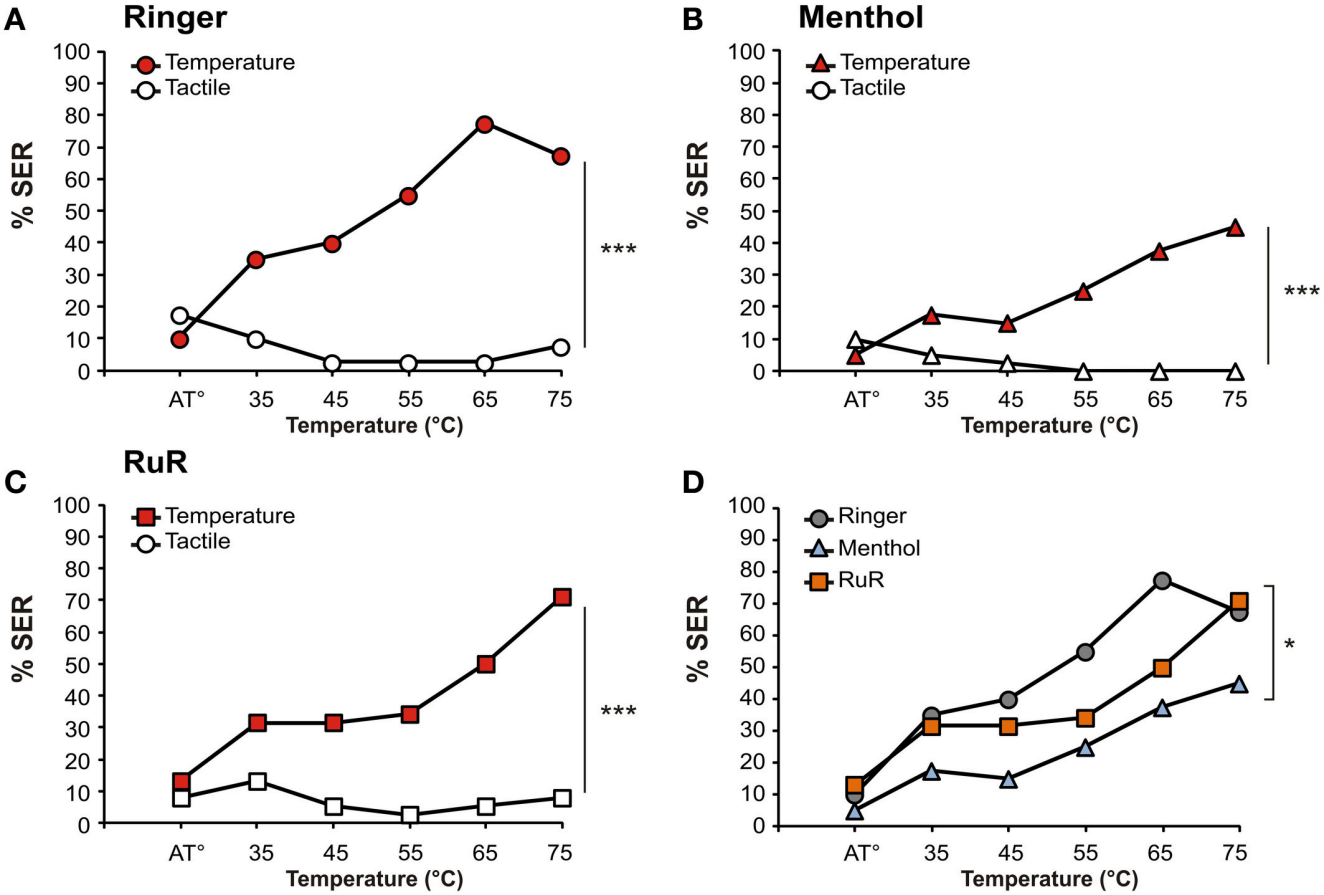
**Effect of HsTRPA inhibitors on thermal responsiveness**. Different groups of bees were injected with Ringer as control (**A**) or with an HsTRPA inhibitor, either menthol (5 mM; **B**) or ruthenium red (RuR, 1 mM; **C**) SER was measured in response to increasing temperatures (red) alternated with tactile controls (white). **(D)** Comparison of thermal response curves among the three groups (Ringer: gray circles; menthol: light blue triangles; RuR: orange squares). Both inhibitors decreased heat responsiveness (^*^p < 0.05; ^***^p < 0.001).

Thus, HsTRPA inhibitors appear to inhibit SER to heat. We next aimed to confirm and expand this result by characterizing the impact of HsTRPA inhibitors on thermal sensitivity along an increasing temperature gradient, as usually tested for measuring bees' aversive responsiveness (Junca et al., 2014; Junca et al. in preparation). Bees were thus injected with the higher dose of each inhibitor or with Ringer, as above, but were then subjected to a series of thermal stimulations at increasing temperatures on the mouthparts alternated with tactile controls (Figures 7A–C). All stimulations were applied at 10 min intervals.

Bees' SER increased significantly with increasing temperature in all three groups (RM-ANOVA, trial effect: Ringer: *n* = 40, *F*_(5, 195)_ = 21.6, *p* < 0.001; RuR: *n* = 38, *F*_(5, 185)_ = 10.8, *p* < 0.001; menthol: *n* = 40, *F*_(5, 195)_ = 9.84, *p* < 0.001). By contrast, responses to alternated tactile stimuli did not increase, and even decreased in the Ringer group, throughout the experiment (RM-ANOVA: ringer: *F*_(5, 195)_ = 2.46, *p* < 0.05; RuR: *F*_(5, 185)_ = 1.22, NS; menthol: *F*_(5, 195)_ = 1.05, NS). Accordingly, in all three groups, responses to the temperature stimulus evolved differently from those triggered by tactile controls (RM-ANOVA, stimulus × trial interaction: Ringer: *F*_(5, 195)_ = 24.6, *p* < 0.001; RuR: *F*_(5, 185)_ = 10.2, *p* < 0.001; menthol: *F*_(5, 195)_ = 9.17, *p* < 0.001]. However, responses to heat were significantly different in the three groups (Figure 7D, RM-ANOVA, stimulus effect: *F*_(2, 115)_ = 5.47, *p* < 0.01; stimulus × trial interaction: *F*_(10, 575)_ = 2.03, *p* < 0.05). In particular, weaker responses were observed in the RuR- and menthol-injected groups compared to the Ringer control (RM-ANOVA, stimulus × trial interaction, Ringer/RuR: *F*_(5, 380)_ = 2.59, *p* < 0.05; Ringer/menthol: *F*_(5, 390)_ = 2.78, *p* < 0.05). No difference appeared between the groups injected with HsTRPA inhibitors (RuR/menthol: *F*_(5, 380)_ = 0.73, NS). Lastly, no difference appeared among groups in the responses to the tactile controls (RM-ANOVA, stimulus effect: *F*_(2, 115)_ = 1.29, NS; stimulus × trial interaction: *F*_(10, 575)_ = 0.74, NS).

**Figure 8:**
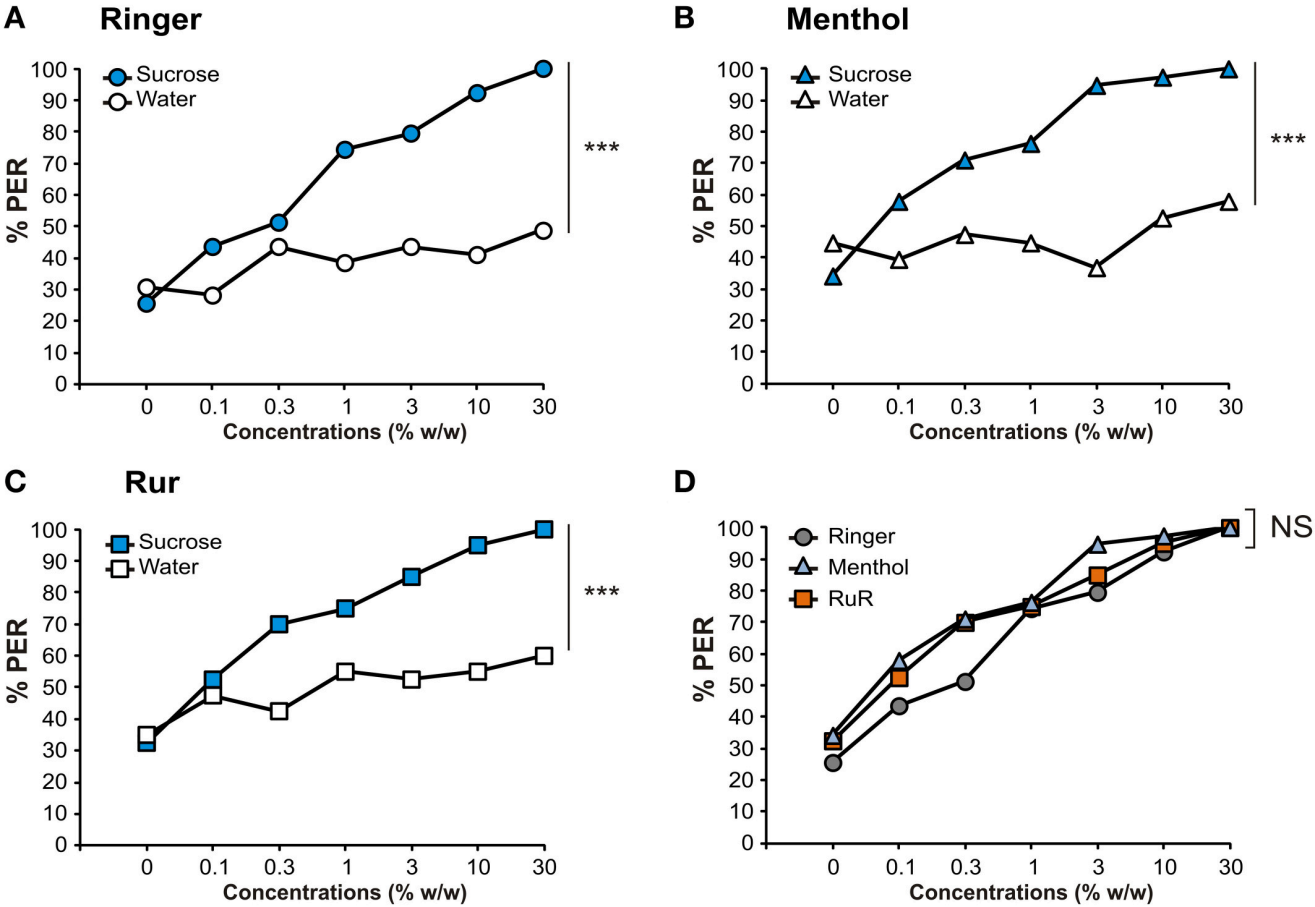
**Effect of HsTRPA inhibitors on sucrose responsiveness**. Different groups of bees were injected with Ringer as control **(A)** or with a HsTRPA inhibitor, either **(B)** menthol (5 mM) or **(C)** Ruthenium red (RuR, 1 mM). Proboscis extension responses (PER) were measured in response to sucrose solutions at increasing concentrations (blue) alternated with water controls (white). **(D)** Comparison of sucrose response curves among the three groups (Ringer: gray circles; menthol: light blue triangles; RuR: orange squares). Inhibitor injections did not impact sucrose responsiveness (NS: Non Significant; ^***^p < 0.001).

The previous experiment confirmed that HsTRPA inhibitors affect thermal responsiveness measured by means of SER. Most probably, this result is due to the effect of the inhibitors on HsTRPA receptors. However, theoretically, it could also be due to a non-specific detrimental effect of the drugs on the bees' physiological state, even though no such effect was apparent by simple observation. In the next experiment, we thus checked the possible effect of HsTRPA inhibitors in another context and another hedonic modality—the appetitive modality. To this end, we measured bees' PER in a typical sucrose responsiveness protocol (Scheiner et al., 2004). After Ringer or HsTRPA inhibitor injections as above, bees were thus subjected to a series of stimulations on the antennae with sucrose solutions at increasing concentrations alternated with water controls (Figures 8A–C). All stimulations were applied at 10 min intervals.

Bees' PER increased significantly with increasing sucrose concentrations in all three groups (RM-ANOVA, trial effect: Ringer: *n* = 39, *F*_(6, 228)_ = 21.9, *p* < 0.001; RuR: *n* = 38, *F*_(6, 234)_ = 24.1, *p* < 0.001; menthol: *n* = 40, *F*_(6, 222)_ = 21.9, *p* < 0.001). Responses to the control water stimulations remained stable for Ringer and menthol but slightly increased for RuR (ringer: *F*_(6, 228)_ = 1.63, NS; RuR: *F*_(6, 234)_ = 2.20, *p* < 0.05; menthol: *F*_(6, 222)_ = 1.45, NS). In all groups, sucrose responses evolved differently from responses to water controls (RM-ANOVA, stimulus × trial interaction: Ringer: *F*_(6, 228)_ = 8.03, *p* < 0.001; RuR: *F*_(6, 234)_ = 6.50, *p* < 0.001; menthol: *F*_(6, 222)_ = 10.0, *p* < 0.001). However, responses evolved similarly in the three groups both for sucrose stimulations (Figure 8D; RM-ANOVA, stimulus effect: *F*_(2, 114)_ = 1.44, NS; stimulus × trial interaction: *F*_(12, 684)_ = 0.68, NS) and for the water controls (stimulus effect: *F*_(2, 114)_ = 0.85, NS; stimulus × trial interaction, *F*_(12, 684)_ = 0.68, NS). We conclude that HsTRPA inhibitors have no effect on bees' PER responses to sucrose, suggesting that their effect on heat-evoked SER is not due to a general behavioral impairment.

## Discussion

Our study provides the first heat sensitivity map of the honeybee, measured using heat-induced SER. This map reveals that responses are symmetrical between body sides, that body structures are more sensitive than the appendages and it shows a gradual decrease in thermal sensitivity from the head to the abdomen. We then demonstrated that heat application does not need to be located on specific structures (mouthparts, antennae or protarsi) to serve as an aversive US in SER conditioning. Indeed, bees learned successfully when the US was provided on the vertex or on the ventral abdomen (3-4 sternites). Lastly, we observed that HsTRPA activators (AITC, CA, camphor) applied topically on the bees' mouthparts did not easily induce SER (only AITC at the higher dose) whereas inhibitor injections (RuR, menthol) significantly decreased SER to heat. This impact of HsTRPA inhibitors was specific of SER to heat, since no effect was observed on PER responses to sucrose.

### Thermal body map

We observed that bees' heat sensitivity, as measured by the induced SER, varied among body structures. Control tactile stimulations also led to variations in responses among body structures but on a much smaller scale compared to heat-triggered responses. Thus, most of the observed SER were due to heat application. The map showed clearly that heat detection is a general phenomenon and is not restricted to a few dedicated sensory structures, like the antennae, mouthparts or tarsi (Junca et al., 2014). A possible explanation for this observation may originate from the high temperature (65°C) used for thermal stimulation, which may have induced activation of nociceptive pathways responsible for preserving the animals' physical integrity. Such system should be differentiated from fine-tuned thermosensory pathways which detect temperatures in the physiological range and employ dedicated thermosensitive sensilla (coelocapitular sensilla) on the bee antenna (Lacher, 1964; Yokohari et al., 1982; Yokohari, 1983). The existence of nociceptive pathways in insects has been recently demonstrated in Drosophila larvae, in which the detection and avoidance of noxious heat, bright light, or strong mechanical stimuli is operated by class IV multidendritic neurons that express a range of nocisensor proteins (Im and Galko, 2012). These neurons extend their dendrites within the derma and are widely distributed along the body surface (Hwang et al., 2007). Although, strongly remodeled, they survive through metamorphosis and may play a similar role in adults (Kuo et al., 2005; Shimono et al., 2009). The wide field heat sensitivity we have found in this study would fit with the existence of an analogous neuron family in honeybees. To this day, however, they have not yet been described. Alternately, thermosensation may also involve some of the many sensory hairs present on the bee body. Only a few structures of the bee body did not elicit more SER when they were thermally stimulated than with the tactile control: the tip of the abdomen and the distal part of the forewings. A possible lack of nociceptive neurons in the wings may explain this observation. At the tip of the abdomen, it would seem rather unlikely that nocisensor neurons are utterly absent. Rather, the proximity between the heat stimulus and the sting chamber might have prevented any sting extension, the animal attempting to avoid any internal injuries.

Responses to heat were compared among body parts. First, we did not find any lateralization bias on the paired appendages. The opposite would have been surprising. Indeed, organisms expressing such an asymmetrical perception would suffer from obvious disadvantages (Corballis, 1998). The physical world is indifferent to left and right, and any lateralized deficit might leave an animal vulnerable to attacks on one side or unable to attack prey or competitors appearing on one side (Vallortigara and Rogers, 2005). Second, peripheral structures appeared less sensitive than body structures. This difference was mostly due to a lower sensitivity of appendages to tactile stimuli, which could be related to the fact that appendages are more likely to come in contact with mechanical substrates than the body. Lastly, we observed a gradient of decreasing thermal responsiveness from the head to the abdomen. The brain located in the head capsule contains neuropils essential for processing and integrating information from many sensory modalities (gustatory, olfactory, visual, tactile, etc) as well as for motor control, navigation, learning, and memory processes among others (Menzel, 1999, 2012). Therefore, physical integrity of the head is crucial for bees to be able to assess their environment and exhibit adapted behaviors, and noxious simulations located close to the head should trigger stronger responses.

### SER learning on the vertex and the ventral abdomen

**Figure 9:**
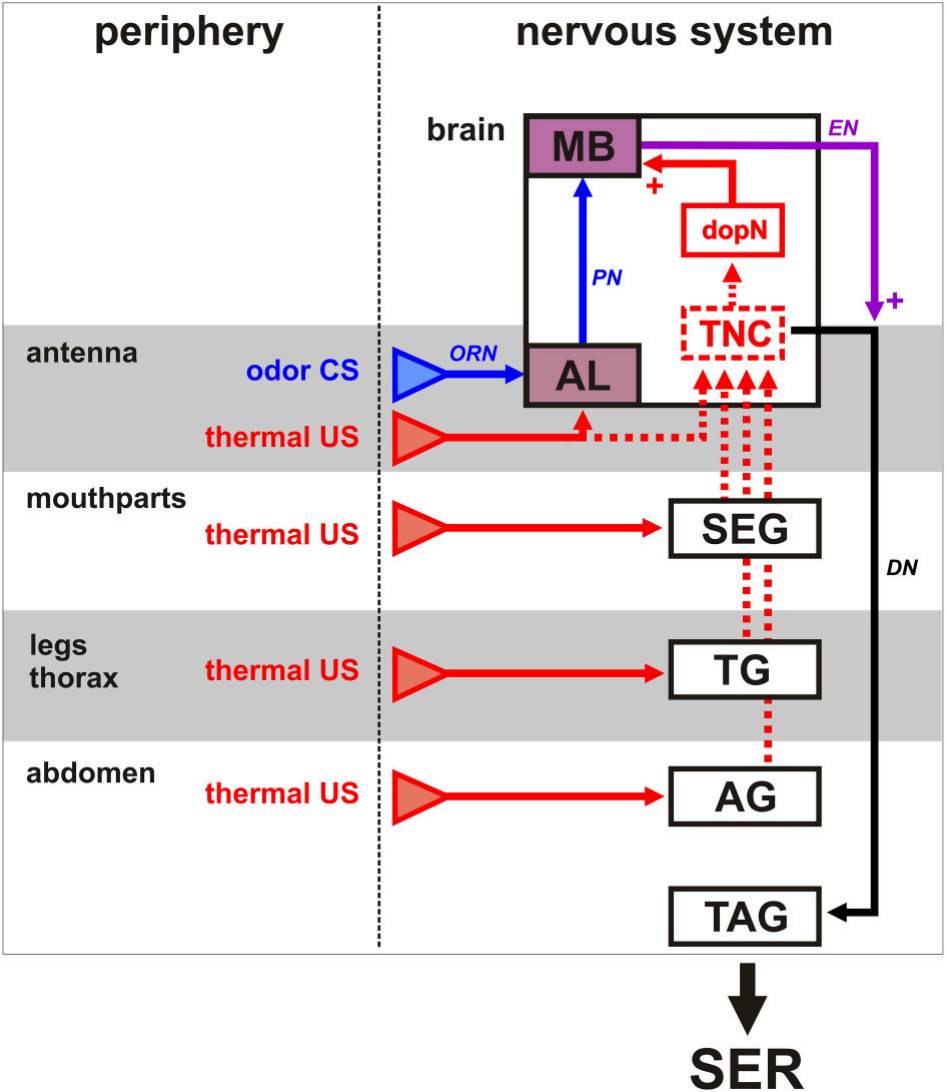
**Working model of aversive olfactory conditioning of SER using a thermal US**. Putative pathways involved in **(A)** the expression of SER after thermal stimulation, **(B)** the acquired SER after learning a CS-US association, are shown. **(A)** At the periphery, stimulation of the different structures with a high temperature is thought to activate thermosensitive neurons (possibly class IV multidendritic neurons), which would first project to the respective relays on the ventral nerve cord, the subesophageal ganglion (SEG), thoracic ganglia (TG), or abdominal ganglia (AG). As a second step, interneurons would project to a thermal/nociceptive center (TNC) in the brain. Antennal thermal stimulation induces activity in the antennal lobe (AL) but possibly also activates the TNC. Activation of this center would stimulate premotor descending neurons (DN) which would in turn trigger stinging motor patterns in the terminal abdominal ganglion (TAG), producing SER (Ogawa et al., 1995). **(B)** Olfactory learning: odorants are detected on the antenna by olfactory receptor neurons (ORNs) projecting to the AL. Then information is prominently conveyed to the mushroom bodies (MB) by projection neurons (PN). Activation of dopaminergic neurons (dopN) by the TNC would inform the olfactory pathway of the aversive thermal reinforcement. Associative plasticity at the level of MB extrinsic neurons (EN) feeding onto the sting premotor descending neurons would allow the CS to elicit SER after learning.

In a previous study, we demonstrated that thermal SER conditioning is successful with a heat US on the mouthparts, the antennae and the tarsi of the forelegs (Junca et al., 2014). Such structures are well known sensory organs (Hammer, 1993; de Brito Sanchez et al., 2008; Giurfa and Sandoz, 2012; Jung et al., 2015). We show here that heat stimulation on body structures that are not dedicated sensory organs (vertex, ventral abdomen) can also act as US in SER conditioning. This observation supports our current putative neural model of thermal aversive conditioning in honeybees (Figure 9). Associative learning relies on the convergence of CS and US information at one or several locations in the brain. The olfactory (CS) pathway is well known in bees (Menzel, 1999; Giurfa, 2007; Sandoz, 2011): axons of olfactory receptor neurons (ORN) located on each antenna project to the antennal lobes (AL) where they synapse with approximately 4000 local interneurons (not shown) and 800 projection neurons (PN). Projection neurons then convey processed information to higher-order brain structures, the mushroom bodies (MB) and the lateral horn (not shown). For aversive learning, the US pathway is mostly unknown, but our results may provide some new clues. Except for the case in which an antenna heat US is used (Junca et al., 2014), and for which thermo-sensory neurons from the antenna are thought to project to the antennal lobe (Yokohari, 1983; Nishino et al., 2009), all other heat stimulations probably rely on thermal detection by the above-mentioned putative multidendritic neurons. It is unlikely that this information also projects to the antennal lobe. Rather, it can be expected from neuroanatomical work in other insects (for instance on the mechanosensory system, Pflüger et al., 1988; Newland and Burrows, 1997) that such putative thermo-sensitive/nociceptive neurons would first project to the respective ganglia of the ventral nerve cord, i.e., to sub-esophageal, thoracic or abdominal ganglia depending on the location of the stimulation (SEG, TG, and AG in Figure 9). From there, information could be conveyed by ascending interneurons toward the brain, possibly to a thermal/nociceptive integration center (TNC in Figure 9), as suggested by several observations. In the Asian bee *Apis cerana*, immediate early gene (Acks) expression mapping showed that exposure to a high temperature (46°C) induces neural activity in several brain regions: within the mushroom body, intrinsic neurons (class I and II Kenyon cells), and in a region of the protocerebrum located between the dorsal and the optic lobe (Ugajin et al., 2012). Thus, stimulation with a high temperature presumably induces activity in one thermo-sensitive center and in the mushroom bodies, a well-known multimodal integration and association center of the bee brain. Our working hypothesis is that neurons from the putative thermo-sensory center could then activate aversive reinforcement circuits, which would converge with the olfactory pathway and induce learning-associated plasticity, in particular in the mushroom bodies. Previous work on SER conditioning indicated that dopaminergic neurons (dopN in Figure 9) are involved in aversive reinforcement, because pharmacological blockade of dopamine receptors disrupts aversive learning (Vergoz et al., 2007). Dopamine neurotransmission is also necessary for aversive learning in other insects (Drosophila, Schwarzel et al., 2003; Schroll et al., 2006; crickets, Unoki et al., 2005). The bee brain contains a complex arrangement of many dopamine-immunoreactive neurons (Schäfer and Rehder, 1989; Schürmann et al., 1989). Among dopamine neurons, three clusters are especially interesting as they contain processes that project to the mushroom body calyces and lobes (especially the α-lobe), and may thus provide aversive reinforcement information (Tedjakumala and Giurfa, 2013). Co-activation of CS and US pathways could modify the strength of synapses between the specific Kenyon cells representing the learned odorant and mushroom body extrinsic neurons (EN in Figure 9) feeding onto the sting extension premotor system. After learning, presentation of the odor CS alone would trigger SER thanks to this modification. Further work is needed to confirm the different putative elements of this working model. The present study started this task by evaluating potential receptors detecting temperature at the periphery (see below).

### Putative involvement of HsTRPA in heat perception

We assessed the possible involvement of HsTRPA in heat-triggered SER using topical applications of activators and injections of inhibitors. We observed that topical application of HsTRPA activators is not sufficient for triggering SER, except when a very high concentration (100 mM) of AITC was used as stimulus. This result might appear surprising since all three tested drugs were potent activators of the channel *in vitro* (Kohno et al., 2010). However, if thermosensation is carried out by a similar class of class IV multidendritic neurons as in Drosophila (Im and Galko, 2012), it is likely that the thermal channels are located in the epidermis, i.e., below the cuticle, so that direct contact of the activators with the channel is not possible, or at least difficult. Heat could diffuse through the cuticle to activate the channel, but chemical activators would not. In our view, therefore, this result does not invalidate a potential role of HsTRPA in thermal sensitivity and nociception in bees. Concerning the SER increase observed with AITC stimulation, we cannot be sure at this stage that it is not related to a possible aversive gustatory effect of this compound when presented to the mouthparts, because AITC was found to inhibit PER responses when added to sucrose solution (Kohno et al., 2010). However, in the same study, the effect of AITC was reversed by RuR, suggesting a possible involvement of HsTRPA. Until now no SER in response to bitter or repellent gustatory stimuli has been reported. It will be necessary to test the effect on SER of AITC application on other locations of the bee body, while also checking if known aversive gustatory stimuli (salt or bitter compounds) can trigger SER when applied on the mouthparts. This will be addressed in more details in the future.

Injections of HsTRPA inhibitors produced significant blocking of SER in response to heat. This effect is similar to the reversal of the suppression of PER by heat in previous work (Kohno et al., 2010). In this study, heating a sucrose solution to 70°C was found to decrease bees' PER to sucrose, compared to an unheated solution. Both RuR and menthol restored normal PER responses in the presence of the heated sucrose solution, presumably by blocking HsTRPA activity (Kohno et al., 2010). The effective inhibitor concentrations in our study were about 10 times higher than the concentrations that significantly modified bees' warmth (36.5°C) avoidance in a thermal gradient (0.1 mM RuR and 0.5 mM menthol, Kohno et al., 2010). It is possible that inhibition of the highly-sensitive stinging response requires higher inhibitor concentrations (i.e., more general blocking of HsTRPA channels) than a fine-tuned behavior like warmth avoidance. Alternately, the mode of injection performed in the two studies (ocellar injection in the present study, injection between the antennae in Kohno et al., 2010) might be involved. Performing both experiments in the same conditions may clarify this question. As a control for the effect of the drugs on thermally-induced SER, we tested the effective concentrations on bees' PER to sucrose and found that neither RuR nor menthol had any effect. If indeed both compounds act on HsTRPA, as we suppose, such a result could have been expected since responses to sucrose are mediated by dedicated gustatory receptors, mostly AmGr1 (Jung et al., 2015). This confirms, however, that RuR and menthol did not reduce SER to heat through a non-specific effect on bees' general responsiveness to stimuli, but rather specifically inhibited their responses to heat.

For the moment, we need to remain cautious about the involvement of HsTRPA in bees' heat sensitivity, as a neuropharmalogical approach alone is not sufficient for demonstrating the role of this TRP channel *per se*. Indeed, the chemical activators and inhibitors we have used are also known to be inhibitors/activators of other members of the TRP family in other species. For instance, in mammals, menthol is able to activate TRPM8 (cold, Behrendt et al., 2004), while RuR is a non-specific inhibitor of TRPM8 (Story et al., 2003) and all four TRPV channels (fine temperature deviation to extreme heat, Clapham et al., 2001; Clapham, 2003). It would thus be especially important in the future to use a technique for blocking HsTRPA more specifically, for instance using RNA interference (Farooqui et al., 2003; Louis et al., 2012), especially because bees express other TRP channels. In invertebrates, channels belonging to the TRPA subfamily are more specifically involved in thermal detection (Matsuura et al., 2009). Most prominently, TRPA1 and Painless have been well described in Drosophila and were shown to be crucial for thermal nociception (Tracey et al., 2003; Hamada et al., 2008; Kohno et al., 2010; Neely et al., 2011). In addition, Pyrexia, another TRP channel, plays a significant part in heat detection and tolerance in this species (Lee et al., 2005). The honeybee genome, as that of other Hymenoptera, does not contain any TRPA1 channel. It is thought that *HsTRPA*, which has evolved from the duplication of an ancestral hygrosensor (*Wtrw*), has gained thermoresponsive properties, which may have resulted in the loss of *TRPA1* in Hymenoptera (Matsuura et al., 2009). Consequently, HsTRPA is considered as a prominent thermosensor in bees and our results suggest it is involved in heat sensitivity leading to SER. However, homologs of the Drosophila genes painless and pyrexia have been described in the honey bee genome, and named *AmPain* and *AmPyr* respectively (Matsuura et al., 2009). It would thus be important to evaluate next the possible involvement of these two channels in heat sensitivity and thermal aversive conditioning. Thanks to the thermal sensitivity map we have established, future studies will be able to compare the relative sensitivity of the different body parts with the expression patterns of *AmHsTRPA, AmPain* and *AmPyr* in the bee body. In addition, SER triggered by heat stimulation, coupled to the use of RNA interference will allow testing the involvement of each channel.

In conclusion, this study constitutes a first step for understanding heat perception and aversive SER conditioning in honey bees. Our current results suggest that a RuR- and menthol-sensitive thermal receptor, possibly HsTRPA, is involved in heat sensitivity leading to sting extension and may represent the peripheral US detector in our aversive conditioning protocol.

### Conflict of interest statement

The authors declare that the research was conducted in the absence of any commercial or financial relationships that could be construed as a potential conflict of interest.
